# Investigate the Dosimetric and Potential Clinical Benefits Utilizing Stereotactic Body Radiation Therapy With Simultaneous Integrated Boost Technique for Locally Advanced Pancreatic Cancer: A Comparison Between Photon and Proton Beam Therapy

**DOI:** 10.3389/fonc.2021.747532

**Published:** 2021-09-22

**Authors:** Peilin Liu, Xian-shu Gao, Zishen Wang, Xiaomei Li, Xi Cao, Chenghao Jia, Mu Xie, Feng Lyu, Shiyu Shang, Xuanfeng Ding

**Affiliations:** ^1^ Department of Radiation Oncology, Peking University First Hospital, Beijing, China; ^2^ Department of Radiation Oncology, Hebei Yizhou Tumor Hospital, Zhuozhou, China; ^3^ Department of Oncology, Hebei North University, Shijiazhuang, China; ^4^ Department of Radiation Oncology, Beaumont Health, Proton Beam Therapy Center, Royal Oak, MI, United States

**Keywords:** normal tissue complication probability (NTCP), stereotactic body radiation therapy (SBRT), simultaneous integrated boost (SIB), pancreatic cancer, intensity modulated proton therapy (IMPT), volumetric modulated arc therapy (VMAT)

## Abstract

**Purpose:**

To investigate the potential clinical benefits of using stereotactic body radiation therapy (SBRT) with simultaneous integrated boost (SIB) technique for locally advanced pancreatic cancer (LAPC) among different treatment modalities and planning strategies, including photon and proton.

**Method:**

A total of 19 patients were retrospectively selected in this study: 13 cases with the tumor located in the head of the pancreas and 6 cases with the tumor in the body of the pancreas. SBRT-SIB plans were generated using volumetric modulated arc therapy (VMAT), two-field Intensity Modulated Proton Therapy (IMPT), and three-field IMPT. The IMPT used the robust optimization parameters of ± 3.5% range and 5-mm setup uncertainties. Root-mean-square deviation dose (RMSD) volume histograms were used to evaluate the target coverage robustness quantitatively. Dosimetric metrics based on the dose-volume histogram (DVH), homogeneity index (HI), and normal tissue complication probability (NTCP) were analyzed to evaluate the potential clinical benefits among different planning groups.

**Results:**

With a similar CTV and SIB coverage, two-field IMPT provided a lower maximum dose for the stomach (median: 18.6GyE, p<0.05) and duodenum (median: 32.62GyE, p<0.05) when the target was located in the head of the pancreas compared to VMAT and three-field IMPT. The risks of gastric bleed (3.42%) and grade ≥ 3 GI toxicity (4.55%) were also decreased. However, for the target in the body of the pancreas, VMAT showed a lower maximum dose for the stomach (median 30.93GyE, p<0.05) and toxicity of gastric bleed (median: 8.67%, p<0.05) compared to two-field IMPT and three-field IMPT, while other maximum doses and NTCPs were similar. The RMSD volume histogram (RVH) analysis shows that three-field IMPT provided better robustness for targets but not for OARs. Instead, three-field IMPT increased the Dmean of organs such as the stomach, duodenum, and intestine.

**Conclusion:**

The results indicated that the tumor locations could play a critical role in determining clinical benefits among different treatment modalities. Two-field IMPT could be a better option for LAPC patients whose tumors are located in the head of the pancreas. It provides lower severe toxicity for the stomach and duodenum. Nevertheless, VMAT is preferred for the body with better protection for the possibility of gastric bleed.

## 1 Introduction

Pancreatic cancer is a malignant tumor with a high mortality rate. It is the sixth leading cause of cancer death in China and the fourth leading cause of cancer death in the United States (1, 2). As of today, surgery remains the only treatment to achieve long-term survival. However, most patients are locally advanced and unresectable when first diagnosed (3). For the locally advanced pancreatic cancer (LAPC) patient population, stereotactic body radiation therapy (SBRT) is the first-line treatment recommended by the guidelines, providing better survival than chemotherapy alone or conventional-fraction radiation therapy (CFRT) (4–6). Although RT dosing for SBRT has not been specified in the guidelines, prescription doses of three fractions (total dose 30–45 Gy) or five fractions (total dose 25–45 Gy) have been applied in some clinical trials (6). In order to have better local control of the hypoxic region in the center of the tumor, simultaneous integrated boost (SIB) was proposed by escalating the dose in the central region (7). A stage I clinical trial proved the safety of delivering 36 Gy in three fractions to borderline resectable pancreatic cancer (BRPC), with a 9-Gy SIB to the positive posterior margins (PM) in patients whose tumor was at least 3 mm away from the duodenum (8). However, it is challenging to administrate such high doses (e.g., the biologically effective dose (BED) of 45 Gy is 85.5 Gy) with photon radiotherapy technique, e.g., volumetric modulated arc therapy (VMAT), when the tumor is adjacent to gastrointestinal (GI) tracts such as the stomach and duodenum with photon therapy. Surpassing dose tolerance to these structures could cause gastrointestinal perforation or ulceration, which could be fatal.

On the other hand, with the rapid development of proton beam technology over the last decades, intensity-modulated proton therapy (IMPT) based on the pencil beam scanning technique has shown potential dosimetric advantage and flexibility to improve organs at risk (OARs) sparing with a sharper fall-off of distal dose compared to photon therapy (9). Proton has proven the advantage to diminish acute toxicities in many diseases such as pediatric low-grade glioma, thymic tumor, and locally advanced non-small cell lung cancer (10–12). Previous studies have reported the potential dosimetric advantage to provide a lower dose for the adjacent GI organs in postoperative pancreatic cancer in comparison with VMAT and passive-scattering technique (13). However, due to the range uncertainties, the proton treatment plan normally enlarges the high dose zone at the distal end of the beam angle, in other words, less conformal to the target volume, in order to provide a robust coverage. Since most of the proton beam angles for LAPC were selected posteriorly, avoiding the bowel gas uncertainties (14), the margin taking into account the range uncertainty directly translated into the high dose spill to the GI organs is critical to the pancreatic SBRT. As a result, not all the studies found that proton beam therapy has the potential clinical advantage in the management of LAPC over photon therapy. For example, Thompson’s study showed that with standard fractions, proton showed no dosimetric advantage in treating LAPC (14). Additionally, Raturi showed that the normal tissue complication probability (NTCP) is not statistically different between photon and proton planning groups. However, these studies did not consider the relationship between the OAR sparing, and the target location since the patient-specific geometry plays a key factor in proton planning (15). Additionally, the feasibility of proton SBRT-SIB for pancreatic tumor has not been addressed yet.

Thus, this study performs a quantitative and comprehensive dosimetric study based on the LAPC location and patient geometry to explore the feasibility and potential clinical benefits of utilizing SBRT-SIB among different treatment modalities and proton field arrangement, including VMAT, two-field IMPT, and three-field IMPT. Furthermore, the NTCP model is implemented to investigate the potential clinical benefits among these planning groups. To the best of our knowledge, this is the first investigation that evaluates the SBRT-SIB plan quality by using the NTCP model for LAPC patient population.

## 2 Method and Materials

### 2.1 Patient Section, Target Volume, and OAR Definition

Nineteen patients with LAPC who received 50.4 Gy in 1.8 Gy per fraction using the VMAT technique in our institution between 2016 and 2020 were selected in this study. All data of the 19 patients we used were approved by Peking University First Hospital Ethics Committee. Tumor location, the volume of the clinical target volume (CTV), planning target volume (PTV), and boost area were shown (**Table 1**). The patient groups were divided by the location of the tumor (head: 13 patients, body: 6 patients). Gross tumor volume (GTV) includes primary tumor and clinically apparent lymph nodes but does not include elective nodal regions (16). GTV to CTV uniform expansions of 0.5 cm were based on ESTRO guidelines (16). For photon therapy, PTV was the CTV plus a 0.5-cm uniform margin. The boost area was 1 cm contracted with CTV to avoid extra dose delivered to adjacent OARs (17). All patients were treated with breath-hold technique, controlling motion in order to assess the maximal potential benefit (18).

**Table 1:** Patient characteristics.

Case	Gender (M/F)	Age (years)	Location	Stage	CTV volume (cc)	PTV volume (cc)	Boost area volume (cc)
1	M	57	head	T4N1M0	134.52	221.19	31.60
2	F	71	body	T3N0M0	29.92	61.52	2.49
3	M	71	head	T3N0M0	47.33	93.92	4.47
4	F	61	head	T4N0M0	55.49	104.62	6.05
5	M	85	head	T4N1M0	75.76	136.17	11.91
6	F	72	head	T4N1M0	143.91	247.22	29.38
7	F	67	body	T3N0M0	75.89	138.02	8.91
8	F	74	head	T4N0M0	46.63	92.90	2.64
9	M	64	head	T3N0M0	117.00	214.47	17.01
10	F	53	head	T3N0M0	73.47	135.98	9.28
11	F	27	body	T3N0M0	58.02	109.77	6.10
12	F	87	body	T4N1M0	57.85	107.57	9.24
13	M	53	body	T4N1M0	160.44	271.96	32.59
14	M	59	body	T4N0M0	85.43	147.31	16.15
15	F	64	head	T3N1M0	113.60	202.22	17.94
16	M	69	head	T3N0M0	69.88	126.23	9.73
17	M	67	head	T4N1M0	170.75	264.99	49.14
18	M	39	head	T3N1M0	65.13	124.91	6.38
19	M	61	head	T3N0M0	356.46	585.90	80.31

M, male; F, female; CTV, clinical target volume; PTV, planning target volume.

### 2.2 Treatment Planning

VMAT, robustness optimized two-field IMPT, and three-field IMPT were all generated on Raystation v 7.0 (RaySearch Laboratory AB, Stockholm). VMAT plans were generated using 6-MV beams with two full arcs, delivered by the Varian linear accelerator (Trilogy, Varian Medical System, Inc., Palo Alto, CA). A collapsed-cone convolution superposition (CCC) based algorithm was applied to calculate, and the dose grid used was 0.3*0.3*0.3 cm^3^.

Proton planning uses CTV plus robustness optimization to take into account the setup and range uncertainties. The plan for IMPT-SIB was done using the single field optimization (SFO) method. Considering the sensitivity of proton beams to inhomogeneous materials and adjacent organs at risk, the directions of the two-field proton plan were posterior, right posterior oblique (19). For the three-field proton plan, the other posterior oblique beam angle was chosen. A CTV-based robust optimization was used, and the plan was evaluated using the worst-case scenario perturbed dose with setup uncertainties of ± 5 mm for x, y, z directions and ±3.5% range uncertainties. The dose calculation was done using the Monte Carlo dose calculation. Proton relative biological effectiveness (RBE) was assumed as 1.1 (20).

The prescription dose of the photon and proton was 30GyE/5f for the target and 45GyE/5f for the boost area. In each plan, 95% volume of the target was requested to receive 95% of the prescription dose. All plans V98 of CTV should reach 98% prescription dose at least, and D95 of the boost area should reach the prescription with the maximum dose limited to 107% prescription. All the treatment plan meets the normal tissues constraints, which were as follows: for the stomach, duodenum, and intestine Dmax (0.5 cm^3^)<35 Gy; for the stomach PRV, duodenum PRV, and intestine PRV Dmax (0.5 cm^3^)<38 Gy; for the spinal cord (0.03 cm^3^)<25 Gy, combined kidneys V12<25 Gy (volume that received 12 Gy should be less than 50% of the volume) and liver V12<40 Gy (18).

### 2.3 Planning Quality Evaluation

To evaluate the dose metric of the photon and proton plans, target coverage and OARs were all compared. Besides, HI of the boost area was used to assess the homogeneity of the plan. HI was defined as follows:


$$HI=D_{95}/D_{5},$$

where *D*_95_ represents the minimum dose in 5% of the target volume, and *D*_5_ represents the minimum dose in 95% of the target volume. The closer the value to 1, the better the homogeneity of the target (21).

### 2.4 Evaluation of Proton Radiation Plan Robustness

The plan robustness was evaluated using the worst-case scenario perturbed dose with setup uncertainties of ±5 mm for x, y, z directions and ±3.5% range uncertainties. The root-mean-square deviation doses (RMSD) volume histograms (RVHs) of all 21 scenarios were generated to evaluate plan robustness (22). The two-field IMPT-SIB plan and three-field IMPT-SIB plan were compared relatively with the area under the RVH curve (AUC) (23). The smaller value of the specific structures indicated that the plan had more robustness in the structure.

### 2.5 Evaluation of NTCP

The cumulative physical dose of all plans was exported from TPS and converted into an equivalent dose in 2 Gy per fraction (EQD2). The evaluation of NTCP was performed using the Lyman–Kutcher–Burman (LKB) NTCP model shown in the following equations (24):


(1) $$NTCP=\frac{1}{\sqrt{2\pi }}\int_{-\infty }^{t}e^{-\frac{x^{2}}{2}}dx$$

where


(2) $$t=\frac{EUD-TD_{50}}{mTD_{50}}$$

with


(3) $$EUD={\left(\Sigma_{i}v_{i}D_{i}^{\frac{1}{n}}\right)}^{n}$$

TD_50_ is the tolerance dose with a 50% probability of complications in the organ; EUD is the equivalent uniform dose; *v_i_* is the volume when a uniform dose *D_i_* is received. Besides, the parameters m and n represent the slope of the dose-response curve and the volume dependence of the NTCP, respectively.

All NTCPs were calculated from converted DVH *via* an in-home program on Matlab version R2019b (MathWorks Inc., Natick, MA, USA). The reference LKB-NTCP parameters (n, m, and TD50), the corresponding endpoints, and the L-Q-model α/β parameter in the present work are shown in **Table 2**.

**Table 2:** Reference LKB-NTCP model parameters (n, m, TD_50_), the corresponding endpoints, and the L-Q-model α/β parameter in the present work.

OAR	n		m	TD_50_(Gy)	α/β	Endpoint	Source
Intestine	0.15		0.79	55	4	Diarrhea	Reinartz. et al. (25)
Intestine	0.15		0.16	55	4	Ulceration/perforation	Burman. et al. (26)
Duodenum	0.193		0.51	299.1	4	Grade ≥3 GI toxicity	Holyoake. et al. (27)
Stomach	0.07		0.3	62	4	Gastric bleed	Pan. et al. (28)

LKB-NTCP model, Lyman–Kutcher–Burman model; OAR, organs at risk; TD_50_, the tolerance dose with a 50% probability of complications in the organ.

In this study, we analyzed the NTCP and compared the results between the two patient populations with tumors located in the head and body of the pancreas.

### 2.6 Statistical Analysis

Analyses were all performed using the SPSS version 24.0 software (IBM, Armonk, NY). Dosimetric outcomes and estimated NTCPs were compared by using Friedman’s test and pairwise comparison with Bonferroni correction between VMAT photon plans and two proton-based plans (two-field IMPT and three-field IMPT). Besides, a two-sided Wilcoxon signed-ranked test was used to compare AUC between two-field IMPT and three-field IMPT. P<0.05 was considered statistically significant.

## 3 Results

### 3.1 Planning Quality Evaluation

**Figure 1:**
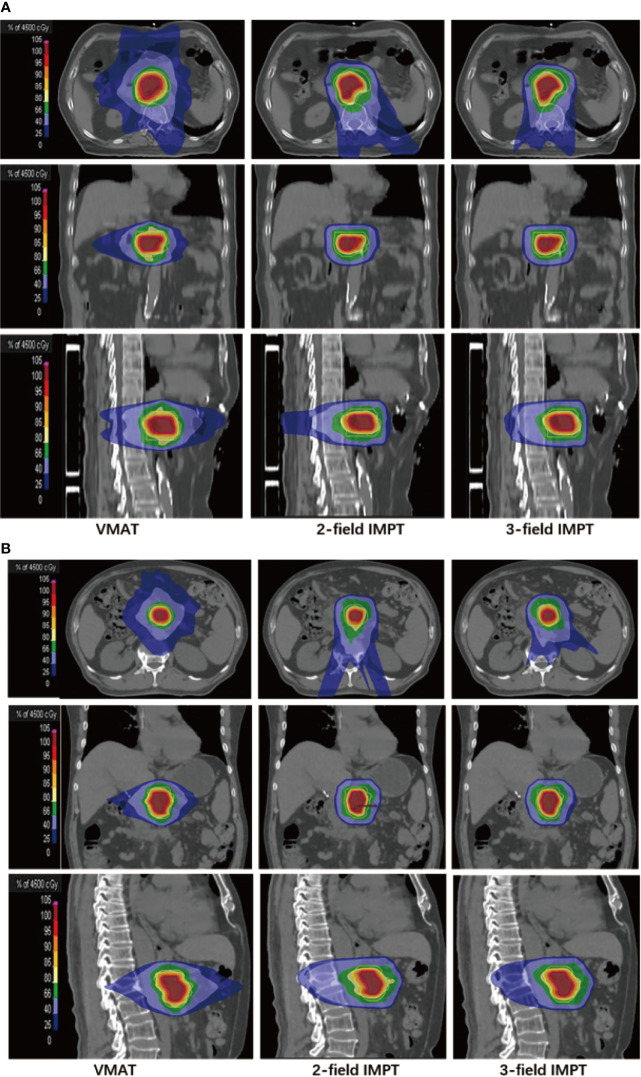
Representative dose contributions for **(A)** tumor located in the head of the pancreas: left (VMAT), middle (two-field IMPT), right (three-field IMPT) patient #5; **(B)** tumor located in the body of the pancreas: left (VMAT), middle (two-field IMPT), right (three-field IMPT) in axial, sagittal, and coronal views, patient #14.

**Figure 2:**
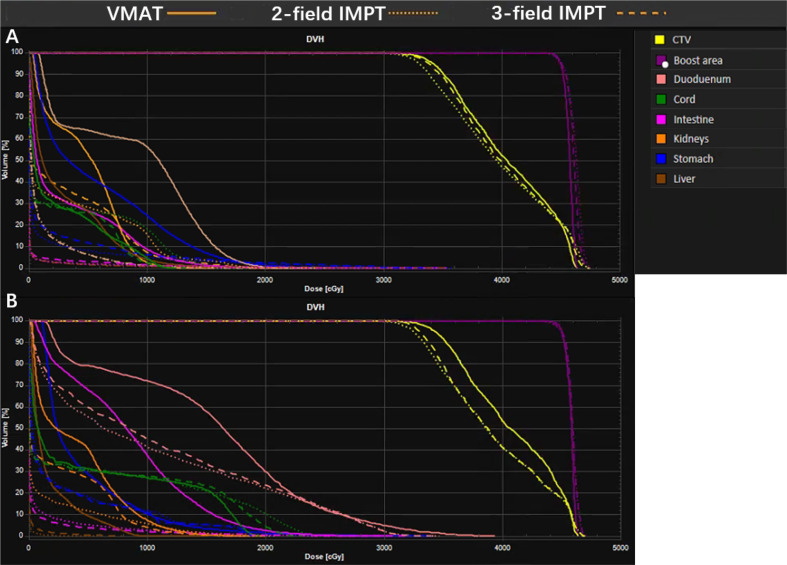
A representative dose volume histogram (DVH) for VMAT, two-field IMPT, and three-field IMPT. **(A)** Tumor located in the head of the pancreas; data are from patient #5; **(B)** tumor located in the body of the pancreas; data are from patient #14.

The detailed summary of target coverage and the dosimetric parameters of OARs are all shown in **Table 3**. Representative dose contributions are displayed in **Figure 1**, for two cases with different locations of the tumors. The corresponding DVHs are also shown in **Figure 2**.

**Table 3:** Dosimetric parameters evaluation.

Dosimetric parameters	Treatment modality			P value		
	VMAT (median and IQR)	2-field IMPT (median and IQR)	3-field IMPT (median and IQR)	VMAT *vs* 2-field IMPT	VMAT vs 3-field IMPT	2-field IMPT *vs* 3-field IMPT
**Boost area**						
**D5(GyE)**						
Head	46.28 (46.26-46.47)	46.70 (46.60-46.86)	46.63 (46.54-46.74)			
Body	46.01 (45.85-46.18)	46.65 (46.46-46.74)	46.67 (46.59-46.78)			
**D95(GyE)**						
Head	45.00 (45.00-45.00)	45.00 (45.00-45.03)	45.00 (45.00-45.00)			
Body	45.00 (45.00-45.00)	45.01 (45.00-45.04)	45.00 (45.00-45.01)			
**HI**						
Head	1.03 (1.02-1.03)	1.04 (1.03-1.04)	1.04 (1.03-1.04)	0.013	0.018	1.000
Body	1.02(1.02-1.03)	1.04 (1.03-1.04)	1.04 (1.04-1.04)	0.250	0.091	1.000
**Mean (GyE)**						
Head	45.77 (45.65-45.83)	45.95 (45.88-46.06)	45.93 (45.87-45.99)			
Body	45.69 (45.53-45.71)	45.99 (45.88-46.00)	45.98 (45.86-46.02)			
**CTV**						
**V98(%)**						
Head	99.92 (99.84-99.98)	99.55 (99.08-99.83)	99.71 (99.57-99.90)			
Body	99.96 (99.95-99.99)	99.94 (99.88-100.00)	100.00 (99.97-100.00)			
**Stomach**						
**Dmax (GyE)**						
Head	21.82 (12.21-29.23)	18.60 (11.50-28.98)	17.85 (11.48-28.97)	0.001	0.003	1.000
Body	30.93 (27.13-32.48)	33.08 (32.30-34.56)	32.36 (31.21-35.06)	0.012	0.063	1.000
**Mean (GyE)**						
Head	4.01 (1.83-6.53)	0.49 (0.26-1.61)	0.70 (0.24-2.28)	<0.001	0.001	1.000
Body	9.87 (6.62-11.30)	3.07 (1.55-6.39)	5.62 (2.42-8.19)	0.007	0.091	1.000
						
**Duodenum**						
**Dmax(GyE)**						
Head	35.39 (34.16-35.87)	32.62 (31.77-32.83)	31.94 (31.24-32.85)	0.001	<0.001	1.000
Body	25.52 (19.46-31.58)	25.26 (14.23-31.25)	26.32 (14.79-31.65)	0.607	0.607	0.607
**Mean(GyE)**						
Head	21.14 (18.18-23.56)	17.23 (14.15-17.79)	16.19 (14.23-18.57)	<0.001	0.005	1.000
Body	6.65 (5.18-8.55)	2.79 (1.55-3.62)	3.35 (1.53-3.89)	0.063	0.012	1.000
**Intestine**						
**Dmax(GyE)**						
Head	33.11 (31.66-35.26)	32.90 (32.06-34.75)	33.52 (32.88-35.02)	0.775	0.775	0.775
Body	32.21 (29.78-34.14)	32.84 (30.83-34.20)	32.94 (32.38-34.35)	0.311	0.311	0.311
**Mean(GyE)**						
Head	8.71 (7.89-10.44)	1.83 (1.07-2.88)	2.57 (1.19-3.70)	<0.001	0.010	0.233
Body	5.05 (3.52-7.79)	1.49 (0.98-2.13)	1.56 (1.12-2.29)	0.003	0.182	0.447
**Kidneys**						
**Mean (GyE)**						
Head	5.67 (4.63-5.83)	4.10 (3.08-5.42)	4.14 (3.75-5.01)	0.199	0.199	0.199
Body	4.11 (3.12-5.13)	3.52 (3.22-3.93)	3.02 (2.49-4.12)	0.607	0.607	0.607
**Liver**						
**Mean (GyE)**						
Head	3.62 (2.39-3.82)	0.48 (0.12-0.98)	0.43 (0.12-1.13)	<0.001	<0.001	1.000
Body	2.89 (2.68-3.85)	0.30 (0.24-0.55)	0.32 (0.24-0.84)	0.007	0.091	1.000
**Spinal Cord**						
**Dmax**						
Head	19.28 (15.46-19.98)	22.25 (19.79-24.39)	21.50 (18.54-23.02)	0.002	0.043	0.980
Body	11.32 (9.37-14.82)	17.82 (16.01-19.30)	18.42 (13.80-18.58)	0.028	0.028	1.000

VMAT, volumetric modulated arc therapy; IMPT, intensity modulated proton therapy; Mean, mean dose; HI, homogeneity index.

#### 3.1.1 Target Coverage

The targets of all treatment methods had reached clinical criteria. As we had observed, all D95 of the boost area reached 45GyE, and two proton plans had the higher D5. VMAT had a slight advantage in HI of the boost area when the tumor was at the head of the pancreas (median 1.03) compared to two-field IMPT (median 1.04, p=0.013) and three-field IMPT (median 1.04, p=0.018). The same HI value of the boost area was obtained for tumors located in the body of the pancreas compared to two-field IMPT and three-field IMPT (all p>0.05). No matter where the tumor was located, the HI of the two kinds of IMPT plans had no statistical significance (p>0.05).

#### 3.1.2 Dose Sparing in OARs

With equivalent target coverage, the remarkable mean dose reductions in most OARs were observed in IMPT planning groups compared to the VMAT (**Table 3**). For the tumors located at the head of the pancreas, the maximum dose of the stomach was decreased from 21.82GyE with VMAT to 18.60GyE with two-field IMPT (p=0.001) and 17.85GyE with three-field IMPT (p=0.063). However, when the tumors were in the body of the pancreas, opposite results were observed. The maximum dose of the stomach in VMAT (median 30.93GyE) was increased with both two-field IMPT (median 33.08GyE, p=0.012) and three-field IMPT (median 32.06GyE, p=0.063).

### 3.2 Evaluation of Proton Radiation Plan Robustness

**Figure 3:**
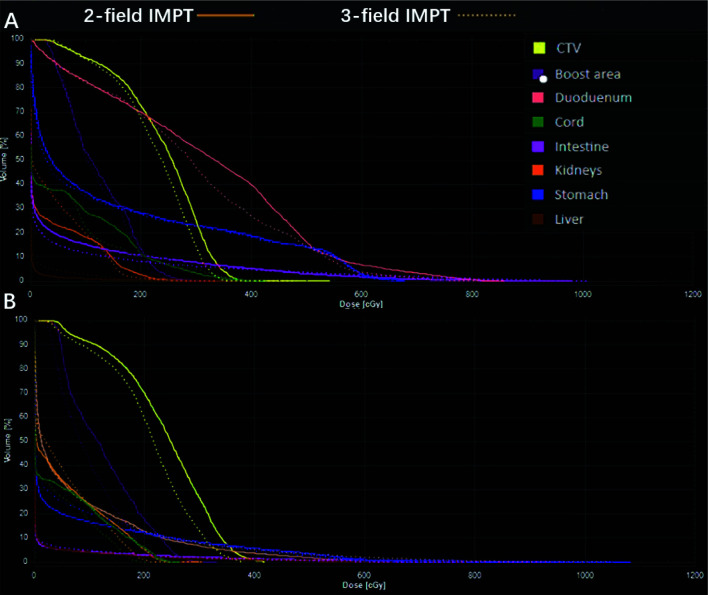
A representative robustness evaluation using RVH: **(A)** tumor located in the head of the pancreas; data are for patient #5; **(B)** tumor located in the body of the pancreas; data are from patient #14.

All the AUC values of target volumes and OARs from the 19 cases were evaluated and are presented in **Table 4**. The typical RVHs are shown in the **Figure 3** with the same patients. The targets showed better robustness when the tumor was at the head of the pancreas when compared to two-field IMPT. The targets include CTV (2.32 in three-field IMPT versus 2.48 in two-field IMPT, p=0.021) and the boost area (1.19 in three-field IMPT versus 1.32 in two-field IMPT, p=0.028). There is no statistical difference among the stomach, duodenum, intestine, liver, and kidneys (p>0.5). Similarly, for tumors located at the body of the pancreas, three-field IMPT improved robustness in CTV (2.32 in three-field IMPT versus 2.47 in two-field IMPT, p=0.028) and the boost area (1.19 in three-field IMPT versus 1.32 in two-field IMPT, p=0.028). There was no statistical significance between two-field IMPT and three-field IMPT in OARs (all p>0.05).

**Table 4:** Proton therapy robustness evaluation.

Structure	Target Location: Head of the pancreas			Target Location: Body of the pancreas		
	2-field IMPT AUC	3-field IMPT AUC	P value	2-field IMPT AUC	3-field IMPT AUC	P value
**Boost area**	1.25 (1.20-1.31)	1.02 (0.97-1.15)	0.021	1.32 (1.27-1.39)	1.19 (1.05-1.35)	0.028
**CTV**	2.48 (2.41-2.58)	2.32 (2.18-2.37)	0.002	2.47 (2.40-2.57)	2.32 (2.14-2.47)	0.028
**Stomach**	0.33 (0.20-0.63)	0.42 (0.21-0.73)	0.504	1.73 (1.06-2.71)	1.76 (1.20-2.64)	0.416
**Duodenum**	3.23 (0.47-1.12)	3.09 (2.94-3.18)	0.506	0.77 (0.75-0.87)	0.82 (0.78-0.89)	0.344
**Intestine**	0.88 (0.47-1.12)	0.92 (0.55-1.11)	0.239	0.55 (0.30-0.76)	0.57 (0.33-0.78)	0.104
**Cords**	0.81 (0.67-1.10)	0.70 (0.61-1.04)	0.009	0.58 (0.53-0.72)	0.52 (0.50-0.57)	0.131
**Liver**	0.10 (0.08-0.29)	0.10 (0.07-0.30)	0.859	0.17 (0.10-0.26)	0.18 (0.10-0.31)	0.343
**Kidneys**	0.73 (0.53-0.83)	0.71 (0.64-0.75)	0.754	0.58 (0.43-0.83)	0.59 (0.39-0.74)	0.917

IMPT, intensity modulated proton therapy; AUC, the area under root-mean-square deviation doses volume histograms curve; CTV, clinical target volume.

### 3.3 NTCP Analysis

**Figure 4:**
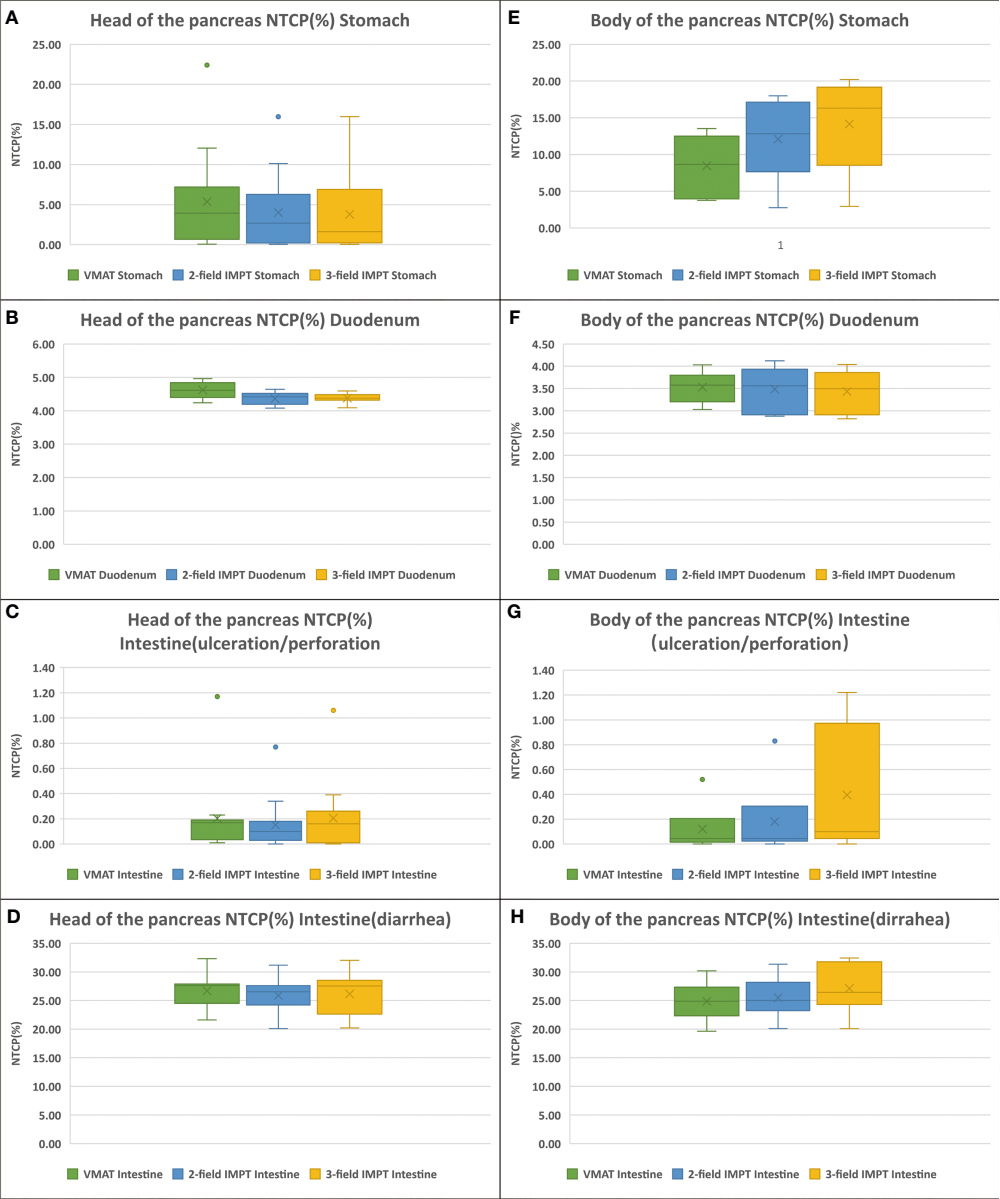
NTCPs comparison of VMAT (green), two-field IMPT (blue), and three-field IMPT (yellow): **(A–D)** (the tumor located in the head of pancreas): ulceration/perforation for stomach, grade ≥ 3 GI toxicity for duodenum, gastric bleed for intestine, and diarrhea for intestine, respectively. **(E–H)** (the tumor located in the head of pancreas): the same order with **(A–D)**.

NTCP values of the stomach, duodenum, and intestine for VMAT, two-field IMPT, and three-field IMPT plans are shown in **Table 5** and **Figure 4**. When the tumor was at the head of the pancreas, both two- and three-field IMPT plans provided lower toxicity of gastric bleed for the stomach (median 2.68%, 1.62%) compared to VMAT (median 3.94%) (p=0.002 and p=0.001, respectively). The risk of grade ≥ 3 GI toxicity of the duodenum was also reduced from a median value of 4.61% with VMAT to 4.42% (two-field IMPT, p<0.001) and 4.38% (three-field IMPT, p=0.001). For the intestine, ulceration/perforation of the three treatment plans were 0.17, 0.10, and 0.18, respectively (all p>0.5). However, when the tumor was located in the body of the pancreas, VMAT provided a lower risk of gastric bleed for the stomach (median 8.67%) with two-field IMPT and three-field IMPT (median 12.83%, 16.32%), although statistical significance was not observed (all p>0.05). The risks of duodenum GI toxicity and ulceration/perforation of the intestine had no statistical significance between VMAT and two-field IMPT (all p>0.05). Besides, VMAT provided better NTCPs of ulceration/perforation (p=0.042) and diarrhea for the intestine (p=0.018). Comparing the two kinds of IMPT plans, all values of NTCP have nonexistent statistical significance (all p>0.05).

**Table 5:** NTCP value.

OAR	Endpoint	NTCP (%) [median and IQR]					
		Head of the pancreas			Body of the pancreas		
		VMAT	2-field IMPT	3-field IMPT	VMAT	2-field IMPT	3-field IMPT
**Stomach**	Gastric bleed	6.73 (0.70-14.83)	3.42 (0.54-11.98)^†^	2.59 (0.49-14.59)^†^	8.67 (5.13-11.34)	12.83 (9.93-16.10)	16.32 (11.58-18.50)
**Duodenum**	Grade ≥3 GI toxicity	4.88 (4.64-5.22)	4.55 (4.28-4.77)^†^	4.58 (4.45-4.71)^†^	3.58 (3.31-3.72)	3.56 (3.02-3.85)	3.50 (3.05-3.75)
**Intestine**	Ulceration/Perforation	0.56 (0.23-1.31)	0.27 (0.10-2.06)	0.35 (0.17-2.12)	0.05 (0.02-0.09)	0.05 (0.03-0.11)	0.10 (0.06-0.70)^†^
	Diarrhea	30.38 (26.52-32.37)	28.66 (26.48-33.88)	29.24 (27.50-33.99)	24.87 (23.47-26.17)	25.02 (24.36-26.7)	26.43 (25.73-30.44)^†^

OAR, organs at risk; IQR, Interquartile range; VMAT, volumetric modulated arc therapy; IMPT, intensity modulated proton therapy.

^†^Compare with VMAT p<0.05.

## 4 Discussion

In the treatment of LAPC, the application of photon therapy is limited because sometimes it fails to deliver a high dose to the target due to the existence of many radiosensitive OARs around. Proton can address this problem due to its unique physical properties. Some studies have shown that proton therapy as part of CRT can achieve satisfying tumor control with low toxicity (29, 30). This study investigated the potential clinical benefits of utilizing the IMPT-SIB technique in the management of the LAPC population through a comprehensive dosimetric comparison among two- and three-field IMPT and VMAT. Under good CTV and boost area dose-coverage obtained from both the IMPT plans and the clinically used photon plans, the results showed that IMPT plans provided lower severe toxicity risks and maximum doses when the tumors were located in the head of the pancreas. However, when the target was located in the body of the pancreas, the clinical benefit of utilizing IMPT diminished due to range overshooting that resulted from the inferior dose conformality. More specifically, for the target located in the pancreatic head, two- or three-field IMPT-SIB reduced the NTCPs of gastric bleed of the stomach and intestinal toxicity of grade 3 and above. For tumors located in the body of the pancreas, VMAT showed lower toxicity of the stomach while other NTCPs were similar. These findings indicated that the model-based approach for patient selection could be an option due to the complicated patient-specific anatomical position (31, 32).

Moreover, we investigated the impact of the beam number and arrangement on the quality of the proton treatment plan. As the degree of freedom increased, the three-field IMPT-SIB plan indeed improved the robustness of targets; but we found that for the OARs, for example, the Dmean of the stomach, intestine, and kidneys was increased due to more entrance dose, raising the chance for low-grade toxicities such as nausea and emesis using three-field IMPT-SIB (33, 34). These findings agreed with the study reported by Stefanowicz et al.; adding one to two beams had no profit in the Dmax and Dmean of OARs with two-field IMPT in pancreatic cancer (17). Adding more fields requires more delivery time and potentially introduces more intrafraction motion, which might undermine the accuracy of treatment delivery (35–37). It makes more fields of IMPT unfavorable or not clinically feasible. However, the recent breakthrough in the rotation arc treatment delivery or call spot-scanning arc therapy (SPArc) introduces more degrees of freedom while improving the treatment delivery efficiency, which is worthy of investigating in the management of LAPC (38). Such technique has shown to be potentially clinically beneficial to various disease sites, including prostate, lung, head, neck, and breast cancer (39).

The application of proton therapy in LAPC using SBRT still faces several challenges. Since organs such as the stomach and the small intestine have significant interfractionation uncertainties such as deformation and gas movement, the accuracy of beam delivery faces difficulties that are critical to the clinical implementation of SBRT (40). Thus, it limits the beam angle selection, which is mostly posterior or posterior oblique. Some portions of the intestine or the stomach are located behind the target, normally receiving a high dose due to the required margin to cover the range uncertainties. Dual-energy CT (DECT) might be able to help in reducing such range uncertainties and make the IMPT plan more conformal compared to the current limitation of using 3.5% range uncertainties (41). Motion mitigation strategies are also critical because the pancreas moves with breathing-induced motion (42). This study is based on the breath-hold technique, which effectively mitigates motion-induced uncertainties. However, such technique has its own limitation. For example, patient training might not work for the person who cannot stand with breath-hold or having an irregular respiratory rhythm that exceedingly prolongs treatment delivery. Gating and tracking would be a direction that we shall investigate in the treatment of LAPC (37). Furthermore, online adaptive MRI-guide radiotherapy will provide the possibility to control the dose distribution and migrate the dose to the OARs with diverse anatomical variations of GI organs such as the gas-filled intestine in the future (43).

Based on the study results, the potential future directions for proton application to LAPC might rely on the following two aspects. On one hand, maximum dose sparing for the stomach and bowel remains a challenge when using proton beam therapy, in which the target space between the intestine and tumors was critical. It indicated that the application of the absorbable hydrogel spacer (TraceIT, Augmenix, Bedford, MA) to separate the head of the pancreas and duodenum could be useful in these cases (44). On the other hand, we should explore the feasibility of different-level dose escalation by increasing the probability of local target control while sparing the OARs utilizing new generation of treatment and planning techniques such as SPArc, minibeams, and functional image-guided dose painting (39, 45, 46).

There are still several potential limitations to our study. The RBE value we used was 1.1, which is the current clinical standard (20). However, recent studies implied that the value of RBE varied depending on the different positions of the SOBP. With the increasing linear energy transfer (LET), the RBE value could reach 1.15–1.7 at the distal edge of the Bragg peak, even 4–6 in the fall-off part (47). For the general anterior beam of the pancreatic cancer plan, the distal edge is generally near the intestine or the stomach. This uncertainty might affect the potential clinical benefits of utilizing the proton beam therapy. The outcomes of proton NTCPs may be reevaluated in the future. To mitigate such RBE uncertainty, the first step is to control better the LET distribution, which could lead to the clinical implementation of the LET optimization algorithm (48, 49).

Besides, the outcomes of our studies rely on the accuracy of the three NTCP models we applied. Please note that the absolute values of △NTCPs for the duodenum (grade ≥ 3 GI toxicity) and the intestine (diarrhea) are small. These differences may not be observed in the clinical outcome study due to the uncertainties and variance of the NTCP model itself. However, the trend of the OAR protection from different treatment modalities and planning strategies might give clinical users a hint to further improve the dosimetric plan quality.

## Conclusion

We have compared the SBRT-SIB plan quality and potential clinical benefits between VMAT, two-field IMPT, and three-field IMPT based on the NTCP model. In the current stage, two-field IMPT is a better option for LAPC patients whose tumor is located in the head. It could provide lower severe toxicity for the stomach and duodenum. However, VMAT is preferred for the body with better protection for the possibility of gastric bleed. Potentially, the model-based approach for patient selection could be an option due to the complicated patient-specific anatomical position.

## Data Availability Statement

The raw data supporting the conclusions of this article will be made available by the authors, without undue reservation.

## Ethics Statement

Written informed consent was obtained from the individual(s) for the publication of any potentially identifiable images or data included in this article.

## Author Contributions

Study conception and design: X-SG, XD, and PLL. Data acquisition: MX, ZW, FL, SYS and CHJ. Data and statistical analysis: PL and XD. Drafting of the manuscript: PL, XC, and XML. Critical editorial and writing contributions: XD, X-SG, and XC. All authors contributed to the article and approved the submitted version.

## Funding 

This work was supported by the China International Medical Foundation (Grant Number: 2019-N-11-07).

## Conflict of Interest

The authors declare that the research was conducted in the absence of any commercial or financial relationships that could be construed as a potential conflict of interest.

## Publisher’s Note

All claims expressed in this article are solely those of the authors and do not necessarily represent those of their affiliated organizations, or those of the publisher, the editors and the reviewers. Any product that may be evaluated in this article, or claim that may be made by its manufacturer, is not guaranteed or endorsed by the publisher.
