# Erratum: Investigate the Dosimetric and Potential Clinical Benefits Utilizing Stereotactic Body Radiation Therapy With Simultaneous Integrated Boost Technique for Locally Advanced Pancreatic Cancer: A Comparison Between Photon and Proton Beam Therapy

**DOI:** 10.3389/fonc.2021.836410

**Published:** 2022-01-04

**Authors:** Frontiers Production Office

**Affiliations:** Frontiers Media SA, Lausanne, Switzerland

**Keywords:** normal tissue complication probability (NTCP), stereotactic body radiation therapy (SBRT), simultaneous integrated boost (SIB), pancreatic cancer, intensity modulated proton therapy (IMPT), volumetric modulated arc therapy (VMAT)

Due to a production error, “Shiyu Shang” was not included as an author in the published article. The corrected affiliations appear below.

Peilin Liu^1^, Xian-shu Gao^1^*, Zishen Wang^2^, Xiaomei Li^1^, Xi Cao^1^, Chenghao Jia^1^, Mu Xie^1^, Feng Lyu^1^, Shiyu Shang^3^ and Xuanfeng Ding^4^*

1 Department of Radiation Oncology, Peking University First Hospital, Beijing, China, 

2 Department of Radiation Oncology, Hebei Yizhou Tumor Hospital, Zhuozhou, China, 

3 Department of Oncology, Hebei North University, Shijiazhuang, China,

4 Department of Radiation Oncology, Beaumont Health, Proton Beam Therapy Center, Royal Oak, MI, United States

The corrected Author Contributions Statement appears below.

“Study conception and design: X-SG, XD, and PLL. Data acquisition: MX, ZW, FL, SYS, and CHJ. Data and statistical analysis: PL and XD. Drafting of the manuscript: PL, XC, and XML. Critical editorial and writing contributions: XD, X-SG, and XC. All authors contributed to the article and approved the submitted version.”

Additionally, author Xi Cao was mistakenly captured as Cao Xi. This error has now been corrected.

The publisher apologizes for these mistakes. The original version of this article has been updated.

